# Ultra-Low Pt Loading Bimetallic PtNi Catalyst on Nano-LTL Zeolite for the Selective Hydrogenation of Halonitrobenzenes

**DOI:** 10.3390/molecules31122042

**Published:** 2026-06-11

**Authors:** Zhen Liu, Guoan Xi, Yin Hu, Wei Chen, Lingling Wang, Xuanye Chen, Fen Zhang

**Affiliations:** 1 Key Laboratory of Jiangxi Province for Environment and Energy Catalysis, Institute of Materials and Intelligent Manufacturing, Jiangxi Academy of Sciences, Nanchang 330096, China; 2 Department of Environment Engineering, Nanchang Hangkong University, Nanchang 330063, China

**Keywords:** p-chloronitrobenzene, selective hydrogenation, PtNi bimetallic catalyst, nano-sized LTL zeolite, mild conditions

## Abstract

The selective hydrogenation of p-chloronitrobenzene (p-CNB) to p-chloroaniline (p-CAN) is of great importance for the production of dyes, pesticides, and pharmaceuticals, but it is often plagued by the undesired hydrodechlorination side reaction. In this work, we report a PtNi bimetallic catalyst supported on nano-sized LTL zeolite (PtNi/Nano-HL) for the selective hydrogenation of p-chloronitrobenzene under mild conditions. The catalyst was systematically characterized by X-ray diffraction (XRD), nitrogen sorption (N_2_ sorption), scanning electron microscopy (SEM), X-ray photoelectron spectroscopy (XPS), and ammonia temperature-programmed desorption (NH_3_-TPD). The results reveal abundant oxygen vacancies (RIR = 0.73) and an optimized distribution of medium–strong acid sites on the catalyst surface, as well as electronic interaction between Pt and Ni, which collectively enhance the catalytic performance. Remarkably, the PtNi/Nano-HL catalyst achieves 100% conversion and over 99% selectivity for p-chloroaniline under ambient conditions (30 °C, 0.1 MPa H_2_) using ethanol as a solvent. Even after 24 recycling runs, it retains 100% conversion and >93% selectivity, demonstrating excellent stability. Moreover, the catalyst requires an extremely low Pt loading (only 0.11 wt%) and exhibits good substrate universality for various substituted nitroarenes. This work provides a promising strategy for designing high-performance bimetallic catalysts on nano-zeolite supports for the selective hydrogenation of halonitrobenzenes.

## 1. Introduction

p-CAN is an important organic intermediate widely used in the synthesis of dyes, pesticides, pharmaceuticals, and other fine chemicals [1]. Its synthesis methods include chemical reduction (Béchamp method) [2], electrochemical reduction [3,4], and catalytic reduction of p-CNB [5]. With the advancement of green chemistry concepts, catalytic hydrogenation has gradually become a more attractive synthetic route due to its high product purity and low pollution. The catalytic hydrogenation of p-CNB is complex, involving parallel and consecutive reactions. According to the reaction network initially proposed by Haber [5], this selective hydrogenation process proceeds via two main pathways: the direct pathway and the condensation pathway. In the direct pathway, p-CNB is first reduced to chloro-nitrosobenzene, then further converted to chlorophenyl-hydroxylamine, and finally to p-CAN. In contrast, the condensation pathway produces azo-benzene intermediates. Notably, a major challenge in this process is the occurrence of hydrodechlorination side reactions, especially during the conversion stage of p-CAN. This side reaction can be attributed to the electron-donating effect of the amino group, which increases the electron density around the C-Cl bond. Therefore, suppressing the hydrodechlorination side reaction is crucial for improving the selectivity for p-CAN.

Noble metals (e.g., Pt [6], Pd [7,8], Au [9], Ru [10]) and non-noble metals (e.g., Ni [11], Co [12], Fe [13], Cu [14]) are widely used as catalysts for the selective hydrogenation of chloronitrobenzene. Among them, Pt-based catalysts have attracted much attention due to their high activity under mild conditions, but they still face the challenge of hydrodechlorination side reactions. To address this issue, a second metal is introduced to modulate the geometric configuration and electronic structure of the active sites, thereby enhancing hydrogenation performance [15,16,17]. For example, Iihama [18] prepared a series of Pt-based bimetallic catalysts supported on SiO_2_ (PtM/SiO_2_) and found that PtZn/SiO_2_ exhibited excellent hydrogenation performance under 1 atm H_2_ and 40 °C, attributed to the formation of electron-rich Pt species that promote hydrogenation of halonitrobenzenes while suppressing hydrodechlorination. Li [19] studied Pt-Cu/C catalysts prepared via two different reduction procedures and observed significant differences in hydrogenation activity while maintaining high selectivity. Recently, Zhu [20] reported that a PtNiCo/C trimetallic catalyst outperformed mono- or bimetallic catalysts in nitrobenzene hydrogenation, with the superior activity originating from the synergistic effect between Pt and (Ni,Co)(OH)_2_. Furthermore, Lu [21] developed a PtPdCoCuNi/SiO_2_ high-entropy alloy catalyst for nitrobenzene hydrogenation, achieving significantly enhanced aniline yield due to the geometric and electronic effects brought about by transition metal alloying. It is worth noting that achieving high selectivity often comes at the cost of catalytic activity [22]. Therefore, it remains challenging to develop efficient catalysts possessing both high activity and high selectivity.

In supported metal catalysts, the choice of support material significantly influences the catalyst structure and hydrogenation performance. Supports are generally divided into two categories: reducible supports (e.g., CeO_2_, TiO_2_, Fe_2_O_3_) and non-reducible supports (e.g., Al_2_O_3_, SiO_2_, MgO). Reducible supports can induce strong metal–support interactions (SMSIs), thereby modulating the geometric and electronic properties of the active sites and improving catalytic performance. For instance, Han [23] studied the effect of different supports on chloronitrobenzene hydrogenation and found that Pt/TiO_2_ exhibited superior catalytic performance due to SMSI. Wang [24] pointed out that, with similar metal particle sizes, electronic effects dominate the hydrogenation behavior of chloronitrobenzene, explaining the higher catalytic activity and chloroaniline selectivity observed on reducible supports. Additionally, the support material itself can adsorb reactants and intermediates, influencing the reaction pathway [25]. Besides commonly used reducible supports, MgF_2_, a less frequently reported support material, has shown potential in the selective hydrogenation of chloronitrobenzene [1,26,27,28]. For example, researchers developed a highly active and selective Pt/MgF_2_ catalyst for the hydrogenation of o-chloronitrobenzene (o-CNB) to o-chloroaniline (o-CAN). In that system, coordinatively unsaturated Mg^2+^ cations act as adsorption sites for o-CNB, while the interaction between Pt and fluoride ions renders Pt electron-deficient.

In this study, we develop a PtNi bimetallic catalyst supported on nano-sized LTL zeolite (PtNi/Nano-HL) for the selective hydrogenation of p-CNB. Systematic characterizations including XRD, N_2_ physisorption, SEM, XPS, and NH_3_-TPD are employed to investigate its physicochemical properties. Compared with previously reported Pt-based catalysts, this work features several advantages: (i) abundant oxygen vacancies and optimized medium–strong acid sites on the catalyst surface, which synergistically promote hydrogenation; (ii) excellent catalytic performance under ambient conditions (30 °C, 0.1 MPa H_2_) with high stability over multiple cycles; (iii) remarkably low Pt loading (0.11 wt%) due to the synergistic effect between PtNi alloy and the zeolite support; and (iv) the first application of nano-sized LTL zeolite as a support for PtNi bimetallic nanoparticles, leveraging its rich pore structure and oxygen vacancies to enhance metal dispersion and electronic regulation. Our results demonstrate that the PtNi/Nano-HL catalyst offers high activity, selectivity, recyclability, and substrate universality, showing great promise for selective hydrogenation of halonitrobenzenes.

## 2. Results and Discussion

### 2.1. Material Characterization

**Figure 1:**
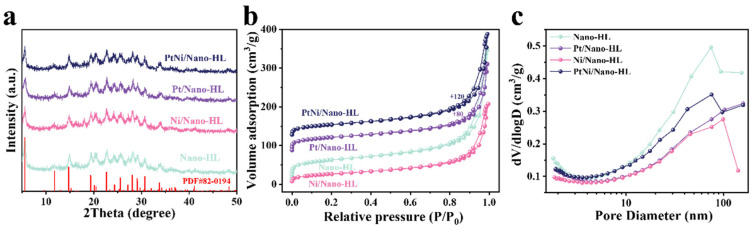
Structural characterizations of different samples. (**a**) XRD patterns, (**b**) N_2_ adsorption–desorption isotherms, and (**c**) non-local density functional theory (NLDFT) pore size distributions.

Figure 1a shows the XRD patterns of the four samples: Nano-HL, Pt/Nano-HL, Ni/Nano-HL, and PtNi/Nano-HL. All samples exhibited distinct diffraction peaks at 2θ = 5.6°, 19.4°, 22.7°, 29.5°, and 30.7°, which are consistent with the characteristic peaks of LTL-type zeolite (JCPDS card No. 82-0194). This zeolite has a space group of P6/mmm (No. 191) with lattice parameters a = b ≈ 18.4 Å, c ≈ 7.5 Å. Notably, due to the low metal loadings and the high dispersion of metal nanoparticles on the support, no diffraction peaks attributable to Pt or Ni species were observed in the XRD patterns.

As shown in Figure 1b, the N_2_ adsorption–desorption isotherms of the four samples all exhibited Type IV isotherms with H4 hysteresis loops. In the low relative pressure region (P/P_0_ < 0.1), a sharp increase in N_2_ adsorption was observed, indicating significant microporous characteristics. When the relative pressure exceeded 0.1, obvious hysteresis loops appeared, confirming the presence of abundant mesopores. From Table 1, the specific surface area and pore volume of the Nano-HL support reached 214 m^2^/g and 0.54 cm^3^/g, respectively. After metal loading, the specific surface area and pore volume of the samples changed to varying degrees, mainly depending on the actual metal loading and their distribution on the support. Combined with inductively coupled plasma optical emission spectrometry (ICP-OES) results, Ni/Nano-HL had a higher actual metal loading, thus its specific surface area and pore volume were lower than those of other samples. In contrast, the PtNi/Nano-HL sample still maintained a relatively high specific surface area (143 m^2^/g), indicating that most of the metal species entered the zeolite channels, with only a small portion loaded on the external surface, leading to moderate reductions in pore structure and surface area. As shown in Figure 1c, the Nano-HL support exhibited a concentrated pore size distribution peak at about 70 nm; PtNi/Nano-HL showed a very similar distribution, while the peak pore size of Ni/Nano-HL increased to about 100 nm. In contrast, the distribution curve of Pt/Nano-HL continuously increased without a distinct peak, indicating a broad pore size distribution and a lack of concentrated mesoporous features. These results further confirm that the distribution of metal species in the zeolite support significantly affects its pore structure and specific surface area.

**Figure 2:**
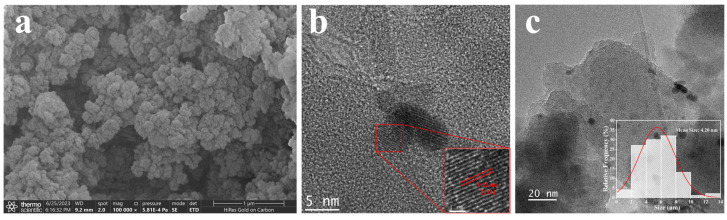
(**a**) SEM images of PtNi/Nano-HL, (**b**) HRTEM image of PtNi nanoparticles in PtNi/Nano-HL, and (**c**) STEM image of PtNi/Nano-HL with particle size distribution.

Figure 2 shows the SEM and high-resolution transmission electron microscopy (HRTEM) images of the Nano-HL system samples. As shown in Figure 2a, the PtNi/Nano-HL catalyst exhibits a typical nanoparticle morphology, providing abundant nucleation sites for the growth of metal nanoparticles on its surface, thereby effectively mitigating particle agglomeration and enhancing catalyst dispersion and stability. The HRTEM image (Figure 2b) shows that the lattice spacing of the PtNi nanoparticles is approximately 0.22 nm, consistent with the (111) plane spacing of PtNi alloy, indicating the formation of a well-defined crystal structure on the support surface [29]. The scanning transmission electron microscopy (STEM) image (Figure 2c) further demonstrates that PtNi nanoparticles are uniformly distributed on the support surface, with a normal distribution of particle sizes and a peak size of 4.2 nm. Such uniform distribution facilitates an increase in active site density and catalytic reaction efficiency. Moreover, the interfacial interaction between the PtNi nanoparticles and the Nano-HL support may have an important influence on the catalytic performance.

For comparison, the monometallic Pt/Nano-L and Ni/Nano-L catalysts were characterized by TEM and STEM (Figures S1 and S2). The Pt nanoparticles exhibit a lattice spacing of 0.20 nm, corresponding to the Pt (111) plane, with a peak particle size of 4.6 nm. The Ni nanoparticles show a lattice spacing of 0.21 nm, matching the Ni (111) plane, and a peak size of 5.5 nm. Both monometallic nanoparticles are uniformly distributed on the Nano-HL support. In contrast, the PtNi alloy nanoparticles (Figure 2) display a smaller average size of 4.2 nm and a lattice spacing of 0.22 nm, consistent with the PtNi (111) plane. These comparative results indicate that the formation of PtNi bimetallic alloy not only reduces the nanoparticle size but also maintains uniform dispersion and well-defined crystallinity, highlighting the structural advantages of the PtNi/Nano-HL system over its monometallic counterparts.

**Figure 3:**
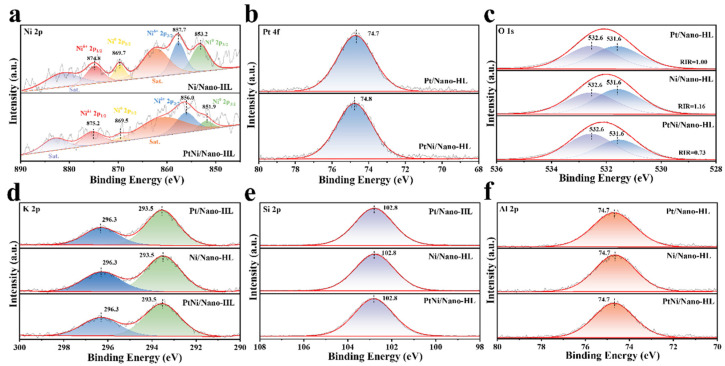
XPS spectra of the Pt/Nano-HL, Ni/Nano-HL, and PtNi/Nano-HL samples for the (**a**) Ni 2p, (**b**) Pt 4f, (**c**) O 1s, (**d**) K 2p, (**e**) Si 2p, and (**f**) Al 2p regions.

Figure 3 shows the XPS spectra of the three catalyst samples. Figure 3a displays the Ni 2p XPS spectra. For the Ni/Nano-HL catalyst, the characteristic peaks at binding energies of 853.2 eV and 869.7 eV are assigned to Ni^0^ species for Ni 2p_3/2_ and Ni 2p_1/2_, respectively; the peaks at 857.7 eV and 874.8 eV are assigned to Ni^δ+^ species for Ni 2p_3/2_ and Ni 2p_1/2_, respectively; and the peaks at 880.9 eV and 862.2 eV are satellite peaks. For the PtNi/Nano-HL catalyst, the characteristic peaks at 851.9 eV and 869.5 eV correspond to Ni^0^ species, while those at 856.0 eV and 875.2 eV correspond to Ni^δ+^ species; the satellite peaks are at 882.8 eV and 860.4 eV [29]. Notably, compared with Ni/Nano-HL, the Ni 2p peaks in the PtNi/Nano-HL sample shift to lower binding energy, indicating an increase in electron cloud density on the Ni surface, likely due to electronic interaction between Pt and Ni. Meanwhile, the Pt 4f XPS spectra (Figure 3b) also display a similar electron transfer phenomenon. In the Pt/Nano-HL catalyst, the main Pt 4f peak is at 74.7 eV, while in the PtNi/Nano-HL catalyst, this peak shifts to 74.8 eV, a positive shift of about 0.1 eV, indicating a decrease in electron cloud density on the Pt surface. This electron transfer not only confirms the formation of a PtNi alloy but also reveals the bimetallic synergy mechanism: the electron cloud density on Pt decreases while that on Ni increases; such electronic structure modulation optimizes the catalyst surface properties, thereby enhancing catalytic activity and selectivity and potentially improving catalyst stability.

To further investigate the surface characteristics of the catalysts, the chemical state of surface oxygen was characterized by XPS to confirm the presence of oxygen vacancies. As shown in Figure 3c, the O 1s spectra can be deconvoluted into two characteristic peaks, corresponding to lattice oxygen and adsorbed oxygen, arranged from low to high binding energy. It should be noted that the appearance of adsorbed oxygen in the O 1s XPS spectrum is considered direct evidence for the formation of oxygen vacancies [30]. Based on this, the relative content of surface oxygen vacancies was quantitatively evaluated by calculating the RIR of lattice oxygen to adsorbed oxygen from the XPS peak areas. The RIR values for the Pt/Nano-HL, Ni/Nano-HL, and PtNi/Nano-HL catalysts are 1.00, 1.16, and 0.73, respectively. The lower RIR value implies that the PtNi/Nano-HL catalyst possesses more abundant oxygen vacancies. This result further suggests that this catalyst benefits not only from the synergy between Pt-Ni bimetals but also from possible strong SMSI. The strong interaction between the PtNi nanoparticles and the zeolite support (Nano-HL) might be attributed to metal-oxygen bonds (e.g., Pt-O-Si or Ni-O-Si) formed at the interface. Such interactions are expected to further enhance catalytic performance by modulating the electronic structure and surface properties of the catalyst. Additionally, as shown in Figure 3d–f, the XPS binding energy changes in K, Si, and Al elements in the three samples were analyzed. The results show that the binding energies of these three elements did not shift significantly in any sample, indicating that K, Si, and Al maintained stable chemical states in the catalytic material. This stability suggests that these elements did not undergo significant chemical environment changes during catalysis, further supporting the overall structural stability of the catalyst.

In summary, XPS analysis not only reveals the presence of abundant oxygen vacancies on the surface of the PtNi/Nano-HL catalyst but also indicates that SMSI may play a key role in enhancing its catalytic performance. Moreover, the stable chemical states of K, Si, and Al in the three catalyst samples further confirm the structural stability of the catalytic materials, thereby providing strong support for their high activity and high selectivity in the hydrogenation of p-CNB.

**Figure 4:**
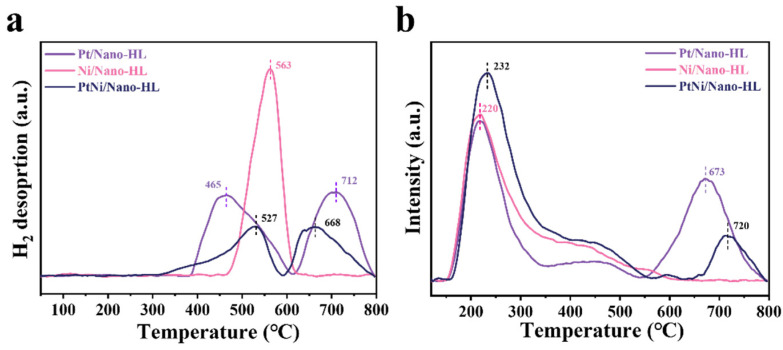
Structural characterization of different samples: (**a**) H_2_-TPD and (**b**) NH_3_-TPD profiles.

As shown in Figure 4a, hydrogen temperature-programmed desorption (H_2_-TPD) was performed to investigate the H_2_ activation ability and hydrogen spillover behavior of the catalysts during the reaction. The results show that the Pt/Nano-HL catalyst exhibits stronger H_2_ activation and hydrogen spillover effects than the other samples. This phenomenon may be related to the content of noble metal Pt: the higher the Pt content, the stronger the H_2_ activation ability and the more significant the hydrogen spillover effect.

From the NH_3_-TPD profiles shown in Figure 4b, three distinct NH_3_ desorption peaks appear in the temperature ranges of <200 °C, 200–400 °C, and >400 °C, corresponding to weak acid, medium–strong acid, and strong acid sites on the catalyst samples, respectively [31]. Specifically, the Pt/Nano-HL catalyst exhibits a desorption peak at 220 °C attributed to its medium–strong acid sites, and a peak at 673 °C attributed to its strong acid sites. When the Pt loading is significantly reduced and Ni metal is introduced (PtNi/Nano-HL catalyst), a desorption peak appears at 232 °C that is higher than those of the other samples, and the desorption peak temperature corresponding to strong acid sites is also higher than that of Pt/Nano-HL. This indicates that the introduction of Ni metal significantly increases the total acid amount of the PtNi/Nano-HL catalyst. This phenomenon can be attributed to the incorporation of some Ni metal into the zeolite cages, causing ion polarization and generating new acid centers, with the acid amount of medium–strong acid sites being mainly enhanced. In contrast, the Ni/Nano-HL catalyst exhibits the fewest weak and medium–strong acid sites, and no strong acid sites were detected. This is due to excessive Ni metal covering many acid sites, leading to a significant reduction in catalyst acidity [32].

### 2.2. Catalytic Performance

To investigate the optimal reaction conditions for the PtNi/Nano-HL catalyst, the effects of various reaction parameters on its catalytic performance in p-CNB hydrogenation were systematically studied. The overall reaction equation is

C_6_H_4_ClNO_2_ + 3H_2_ → C_6_H_6_ClN + 2H_2_O

**Figure 5:**
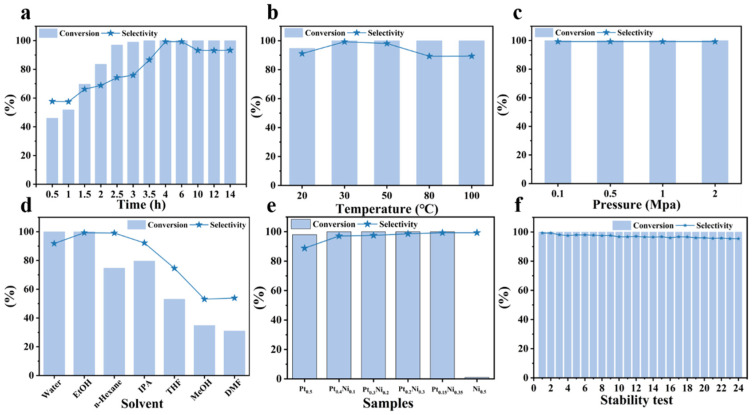
Dependence of the catalytic conversion and product selectivity on various reaction parameters over the PtNi/Nano-HL catalyst in the selective hydrogenation of p-CNB: (**a**) reaction time, (**b**) temperature, (**c**) H_2_ pressure, (**d**) solvent, (**e**) Pt and Ni mass percentages, and (**f**) recycling tests. Reaction conditions (unless varied in the respective panel): p-CNB, 80 mg; catalyst, 50 mg; ethanol, 20 mL; temperature, 30 °C (for panels (**a**,**c**–**f**)); H_2_ pressure, 0.1 MPa (for panels (**a**,**b**,**d**–**f**)); reaction time, 4 h (for panels (**b**–**f**)).

As shown in Figure 5a, the PtNi/Nano-HL catalyst achieved 100% conversion of p-CNB after 3.5 h of reaction, reaching optimal catalytic performance (100% conversion, >99% selectivity for p-CAN) at 4 h. Notably, as the reaction time extended to 7 h, product selectivity declined, which is attributed to over-hydrogenation of p-CNB. However, even when the reaction time was extended to 14 h, the catalyst still maintained excellent selectivity (>93%). Temperature effect studies (Figure 5b) showed that optimal catalytic performance was achieved at room temperature (30 °C). As the reaction temperature increased, the selectivity for p-CAN showed a slight decreasing trend, which is likely due to further dechlorination hydrogenation of p-CAN. Pressure effect experiments (Figure 5c) showed that reaction pressure does not significantly affect the hydrogenation performance of the PtNi/Nano-HL catalyst. Based on green chemistry principles, atmospheric pressure (0.1 MPa) was selected as the optimal reaction pressure.

Solvent screening experiments (Figure 5d) first compared the effects of polar and non-polar solvents, showing that polar solvents are more favorable for the reaction. The lower conversion and selectivity of MeOH relative to EtOH might be tentatively explained by differences in solvation ability and polarity, as well as the possible weak hydrogen-donating role of EtOH via its α-hydrogens, which may facilitate the hydrogenation pathway under mild conditions. MeOH, being more polar and protic, might lead to stronger or less favorable adsorption of substrates or intermediates. Additionally, differences in viscosity or boiling point could also play a role. Further examination of various polar solvents revealed that ethanol as the reaction medium gave the best hydrogenation performance. Catalyst composition optimization experiments (Figure 5e) systematically investigated the effect of different Pt/Ni mass ratios on catalytic performance while keeping the total metal loading (Pt + Ni) at 0.5 wt%. The results in Table 2 showed that as Pt content decreased and Ni content increased, the hydrogenation performance of the catalyst gradually improved, with the PtNi/Nano-HL catalyst exhibiting the best catalytic activity. The monometallic Ni catalyst alone might possess only weak hydrogenation activity at 30 °C, possibly due to its limited ability to activate molecular hydrogen under such mild conditions. In contrast, the presence of Pt in the bimetallic catalyst likely facilitates H_2_ dissociation, and the Pt-Ni interaction may further enhance catalytic performance through electronic or cooperative effects. This indicates that SMSI and bimetallic synergistic effects are key factors for achieving excellent hydrogenation performance.

It is particularly noteworthy that the PtNi/Nano-HL catalyst exhibited excellent cycling stability under optimal reaction conditions. After 24 cycles, the catalyst still maintained 100% conversion and >93% selectivity (Figure 5f). To comprehensively evaluate the substrate universality of this catalyst, the hydrogenation performance for various substituted nitroarenes was further investigated (Table 3). The results show that the PtNi/Nano-HL catalyst exhibits excellent catalytic activity and selectivity for all substrates, confirming its broad application potential in selective hydrogenation of nitro groups. It should be noted that the substrate structure has a significant effect on hydrogenation rate: Entry 1 (p-CNB) required only 4 h for complete conversion, while Entry 2 (o-CNB) and Entry 4 (p-iodonitrobenzene) required 24 h to approach complete conversion. This is mainly attributed to steric hindrance and possible intramolecular interactions (e.g., dipole–dipole repulsion) between the nitro group and the ortho-chlorine atom. Such interactions may twist the nitro group out of the plane of the benzene ring or hinder its adsorption on the catalyst surface, thereby slowing down the hydrogenation process. In contrast, the meta and para isomers experience less steric congestion, allowing faster conversion.

In summary, the PtNi/Nano-HL catalyst not only meets green chemistry principles in reaction conditions but also is economical and efficient in preparation. Its excellent catalytic performance, good cycling stability, and broad substrate applicability make it a promising candidate for selective hydrogenation applications.

## 3. Materials and Methods

### 3.1. Materials

All chemicals and materials, including sodium aluminate (NaAlO_2_), potassium hydroxide (KOH), sodium hydroxide (NaOH), aluminum sulfate octadecahydrate (Al_2_(SO_4_)_3_·18H_2_O), ammonium chloride (NH_4_Cl), chloroplatinic acid hexahydrate (H_2_PtCl_6_·6H_2_O), nickel(II) nitrate hexahydrate (Ni(NO_3_)_2_·6H_2_O), sodium borohydride (NaBH_4_), ethanol, and deionized water, were of analytical grade or higher. LUDOX-40 colloidal silica was purchased from Sigma-Aldrich (St. Louis, MO, USA). Hydrogen gas was used for reduction. Other reagents were obtained from Sinopharm Chemical Reagent Co., Ltd. (Shanghai, China) and Shanghai Aladdin Biochemical Technology Co., Ltd. (Shanghai, China). All materials were used as received without further purification.

### 3.2. Catalyst Preparation

#### 3.2.1. Synthesis of Nano-L Zeolite

Synthesis of L zeolite directing agent: 0.472 g NaAlO_2_ and 3.76 g KOH were dissolved in 10 g H_2_O, stirred for 10 min. Then, 8.4 g LUDOX-40 was slowly added dropwise to the above solution. After stirring for 1 h, 0.24 g NaOH was added, stirred evenly, and aged at room temperature for 3 days to obtain a clear solution.

Synthesis of nano-L zeolite: The specific procedure: 0.99 g KOH and 1.4 g H_2_O were added to 8.9 mL LUDOX-40, stirred for 1 h, then 1.665 g Al_2_(SO_4_)_3_·18H_2_O was added, stirring continued for 1–2 h, followed by dropwise addition of 2.0 mL of the L zeolite directing agent. After stirring at room temperature for 2 h, the resulting synthesis solution was transferred to a Teflon-lined stainless steel autoclave and statically crystallized at 80 °C for 3 days. After crystallization, the product was filtered, washed, and dried to obtain nano-L zeolite.

#### 3.2.2. Synthesis of Nano-HL Zeolite

1 g of nano-L sample was subjected to ion exchange with 50 mL of 1 M NH_4_Cl solution at 80 °C, repeated three times to ensure thorough exchange. The resulting sample was filtered and washed three times with deionized water to obtain NH_4_-type L zeolite. This solid sample was calcined at 550 °C for 5 h to obtain Nano-HL zeolite.

#### 3.2.3. Preparation of Pt/Nano-HL

200 mg of Nano-HL sample was dispersed in 10 mL deionized water, and a certain amount of H_2_PtCl_6_ aqueous solution was added dropwise. The mixture was ultrasonicated for 30 min and then stirred for 24 h. 15 mL of sodium borohydride aqueous solution (containing approximately 0.035 g NaBH_4_) was added to the above mixture, and stirred at room temperature until no bubbles were produced. Subsequently, the mixture was centrifuged, washed twice with deionized water and once with ethanol to obtain a light gray solid. Finally, the solid sample was vacuum-dried at 80 °C overnight to obtain Pt/Nano-HL catalyst.

#### 3.2.4. Preparation of PtNi/Nano-HL

200 mg of Nano-HL sample was dispersed in 10 mL deionized water, and an aqueous solution containing H_2_PtCl_6_ and Ni(NO_3_)_2_·6H_2_O was added dropwise. The mixture was ultrasonicated for 30 min and then stirred for 24 h. The resulting suspension was rotary-evaporated at 90 °C, then dried at 100 °C overnight. Finally, the solid powder was reduced under H_2_ at 400 °C for 2 h to obtain the PtNi/Nano-HL catalyst.

### 3.3. Characterization

The crystalline phase structure of the catalyst samples was determined using a Shimadzu XRD-7000 X-ray diffractometer with a copper target radiation source (Shimadzu Corporation, Kyoto, Japan). The scanning speed was set to 5°/min with a scanning range of 2θ from 5° to 50°. Quantitative elemental analysis was performed on an inductively coupled plasma mass spectrometry (Avio 200 instrument by PerkinElmer, Waltham, MA, USA). Prior to analysis, the powdered catalyst samples were digested in a mixed acid solution of hydrochloric acid and nitric acid (volume ratio 3:1) at 120 °C until a clear and transparent solution was achieved. XPS was employed to investigate the electronic valence states of the elements in the samples. The XPS spectra were acquired using a Thermo Scientific K-Alpha instrument (Thermo Fisher Scientific Inc., Waltham, MA, USA).

To compare the acidic properties of different samples, chemisorption testing of the catalysts was conducted. The chemisorption tests were performed on a TPD AutoChem-2950 instrument, manufactured by Mack (Greensboro, NC, USA). Prior to testing, the powder samples were flaked and sieved to obtain 20–40 mesh catalyst particles. The NH_3_-TPD test procedure was as follows: the temperature was increased to 500 °C under a He atmosphere at a rate of 10 °C/min and maintained for 1 h. The temperature was then decreased to 120 °C, and ammonia was introduced for adsorption for 40 min. After adsorption, the atmosphere was switched back to He, and purging continued for 45 min. Finally, the temperature was increased to 800°C at a rate of 10 °C/min.

The physical adsorption of catalyst powder samples was conducted using an ASAP 2460 instrument from Mack Corporation, USA, to investigate the differences in pore structure. Before testing, the samples were degassed under vacuum at 120 °C for 6 h. N_2_ at 77 K was used as adsorption gas. The specific surface area of the samples was determined by the multi-point BET method, the specific surface area and pore volume of the micropores were assessed using the t-plot method, and the pore size distributions were analyzed using Non-Local Density Functional Theory (NLDFT).

The localization and distribution of metal nanoparticles in the catalyst samples were observed using TEM images captured on a Tecnai G2F30 STWIN TEM (FEI, Waltham, MA, USA) operating at an accelerating voltage of 300 kV. The samples were dispersed in ethanol prior to testing, and then drop-coated onto the surface of microgrid copper mesh. The morphology of catalyst sample was observed using a SIGMA 300 field emission SEM (Zeiss, Oberkochen, Germany) with an accelerating voltage of 3 kV and a beam spot size of 3.0. The samples were dispersed in deionized water, drop-coated onto a silica sheet, and gold-sprayed after the water evaporated.

### 3.4. Hydrogenation of p-CNB

The selective hydrogenation of p-CNB was performed in a 50 mL stainless steel batch reactor. The reaction mixture consisted of 80 mg of p-CNB and 50 mg of catalyst, to which 20 mL of ethanol solvent was added. The reactor was purged with hydrogen three times to displace air, and the reaction was conducted at 500 rpm under the specified time and temperature conditions. After the reaction, the products were quantitatively analyzed using gas chromatography (GC, Agilent 7890B) with a flame ionization detector (FID) and an HP-5 column (30 m × 0.32 mm × 0.25 μm) (Agilent Technologies, Santa Clara, CA, USA). The crucial intermediates were detected and analyzed by gas chromatography-mass spectrometry (GC-MS, Agilent 8890/5977B) with a DB-5MS column (60 m × 0.32 mm × 0.25 μm) (Agilent Technologies, Santa Clara, CA, USA).

## 4. Conclusions

In this study, an efficient and highly selective PtNi/Nano-HL bimetallic catalyst was successfully developed for the selective hydrogenation of halonitrobenzenes by loading PtNi bimetallic nanoparticles onto a nano-sized LTL zeolite support. The catalyst makes full use of the electron transfer effect between Pt and Ni, forming a PtNi alloy and generating significant bimetallic synergy, thereby greatly enhancing hydrogenation activity and selectivity. The nano-LTL zeolite support possesses a high specific surface area (214 m^2^/g) and abundant pore structure (total pore volume 0.54 cm^3^/g), effectively improving the dispersion and stability of PtNi nanoparticles and preventing metal agglomeration. XPS characterization confirmed the presence of abundant oxygen vacancies on the catalyst surface, and SMSI further enhanced catalytic activity; meanwhile, NH_3_-TPD analysis indicated that the appropriate distribution of medium–strong and strong acid sites also significantly promoted hydrogenation performance. Under ambient temperature and pressure, the catalyst achieved 100% conversion and >99% selectivity for p-CNB, with effective suppression of over-hydrogenation. Moreover, the catalyst exhibited excellent catalytic activity and selectivity for various substituted nitroarenes, demonstrating broad substrate applicability. After 24 cycles, the conversion remained 100% and selectivity remained above 93%, reflecting extremely high cycling stability. The mild reaction conditions (low temperature and ambient pressure) align with green chemistry principles and offer significant economic and environmental benefits. This work provides a novel and efficient catalyst design strategy for the hydrogenation of halonitrobenzenes and shows great promise for industrial applications.

## Figures and Tables

**Table 1:** Metal content, specific surface area, and pore volume of the characteristic catalysts.

Sample	Pt Loading ^[a]^ (wt %)	Ni Loading ^[a]^ (wt %)	*S*_BET_^[b]^ (m^2^/g)	*S*_ext_^[c]^ (m^2^/g)	*V*_total_ (cm^3^/g)	*V*_micro_ (cm^3^/g)	*V*_meso_ (cm^3^/g)
Nano-HL	/	/	214	131	0.54	0.04	0.50
Ni/Nano-HL	/	0.58	42	21	0.09	0.01	0.08
Pt/Nano-HL	0.32	/	92	23	0.09	0.03	0.06
PtNi/Nano-HL	0.11	0.40	143	107	0.41	0.02	0.34

^[a]^ Analyzed by ICP. ^[b]^ *S*_BET_ (total surface area) calculated using the Brunauer–Emmett–Teller (BET) equation in the linear range (0.05 < P/P_0_ < 0.3) of the adsorption isotherm. ^[c]^ *S*_ext_ (external surface area), *V*_total_ (total pore volume), *V*_micro_ (micropore volume) and *V*_meso_ (mesopore volume, where *V*_meso_ = *V*_total_ − *V*_micro_) calculated using the *t*-plot method.

**Table 2:** Total metal (Pt + Ni) loading: 0.5 wt%. Reaction conditions: substrate 80 mg, catalyst 50 mg, EtOH 20 mL, temperature 30 °C, H_2_ pressure 0.1 MPa, reaction time 4 h.

Entry	Catalyst	Conv./%	Sel./%
1	Pt_0.5_/Nano-HL	98	88.8
2	Pt_0.4_Ni_0.1_/Nano-HL	100	97.1
3	Pt_0.3_Ni_0.2_/Nano-HL	100	97.5
4	Pt_0.2_Ni_0.3_/Nano-HL	100	98.6
5	Pt_0.15_Ni_0.35_/Nano-HL	100	>99
6	Ni_0.5_/Nano-HL	1	100

**Table 3:** Selective hydrogenation of substituted nitrobenzenes to the corresponding aromatic amines over the PtNi/Nano-HL catalyst. Reaction conditions: reactant, 80 mg; catalyst, 50 mg; ethanol, 20 mL; temperature, 30 °C; H_2_ pressure, 0.1 MPa.

Entry	Substrate	Temp./°C	Time/h	Conv./%	Sel./%
1	** 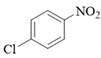 **	30	4	100	>99
2	** 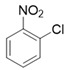 **	30	24	100	>99
3	** 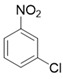 **	30	4	95.6	94.7
4	** 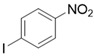 **	30	24	96	84.1
5	** 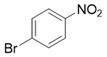 **	30	4	100	96.2
6	** 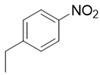 **	30	4	100	96.4
7	** 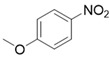 **	30	4	86.6	96.6
8	** 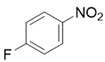 **	30	4	77.3	80.4
9	** 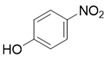 **	30	24	100	94.5
10	** 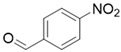 **	30	24	100	73.2
11	** 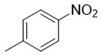 **	30	4	100	94

## Data Availability

The original contributions presented in this study are included in the article. Further inquiries can be directed to the corresponding author.

## Supplementary Materials

The following supporting information can be downloaded at https://www.mdpi.com/article/10.3390/molecules31122042/s1, Figure S1: HRTEM image of Pt nanoparticles in Pt/Nano-HL, and (b) STEM image of Pt/Nano-HL with particle size dis-tribution; Figure S2: (a) HRTEM image of Ni nanoparticles in Ni/Nano-HL, and (b) STEM image of Ni/Nano-HL with particle size distribution.
