# Supplementary material for: Ultra-Low Pt Loading Bimetallic PtNi Catalyst on Nano-LTL Zeolite for the Selective Hydrogenation of Halonitrobenzenes
Source: Molecules. 2026 Jun 11;31(12):2042. doi: 10.3390/molecules31122042 (PMC13305459; doi:10.3390/molecules31122042)
Supplement: Supplementary file 1 [file molecules-31-02042-s001.zip › molecules-4349944-supplementary.pdf]

Supplementary Information

Ultra-Low Pt Loading Bimetallic PtNi Catalyst on Nano-LTL Zeolite for the Selective Hydrogenation of Halonitrobenzenes

Zhen Liu [1,2], Guoan Xi [1,2], Yin Hu [1], Wei Chen [1], Lingling Wang [1], Xuanye Chen [1] and Fen Zhang [1,*]

[1] Key Laboratory of Jiangxi Province for Environment and Energy Catalysis, Institute of Materials and Intelligent Manufacturing, Jiangxi Academy of Sciences, Nanchang 330096, China; 2302085700074@stu.nchu.edu.cn (Z.L.); xiguoan@jxas.ac.cn (G.X.); huyin@jxas.ac.cn (Y.H.); chenwei@jxas.ac.cn (W.C.); wanglingling@jxas.ac.cn (L.W.); chenxuanye@jxas.ac.cn (X.C.)

[2] Department of Environment Engineering, Nanchang Hangkong University, Nanchang 330063, China

* Correspondence: zhangfen@jxas.ac.cn

Figure S1. (a) HRTEM image of Pt nanoparticles in Pt/Nano-HL, and (b) STEM image of Pt/Nano-HL with particle size distribution.

Figure S2. (a) HRTEM image of Ni nanoparticles in Ni/Nano-HL, and (b) STEM image of Ni/Nano-HL with particle size distribution.

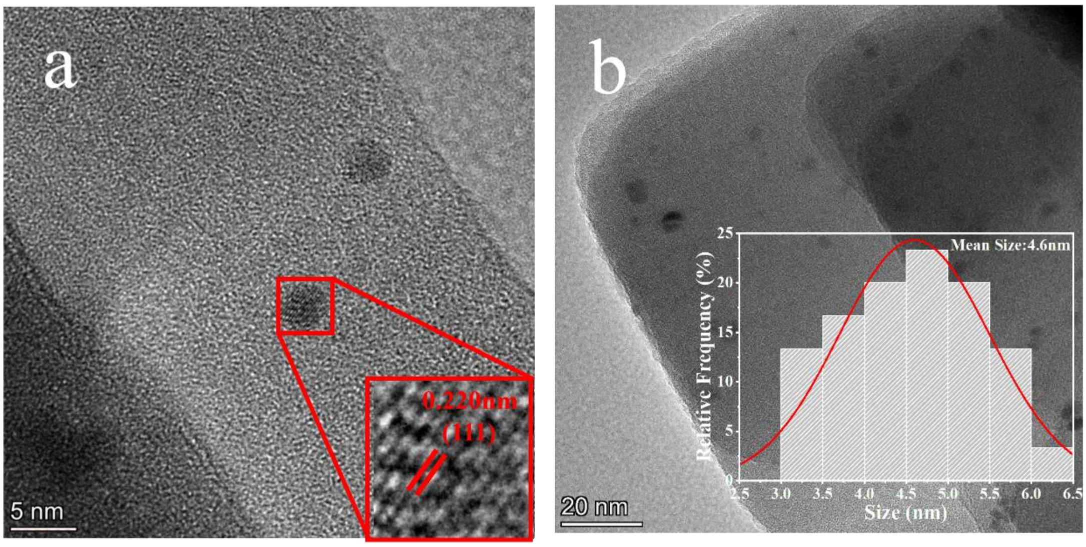

Figure S1. (a) HRTEM image of Pt nanoparticles in Pt/Nano-HL, and (b) STEM image of Pt/Nano-HL with particle size distribution.

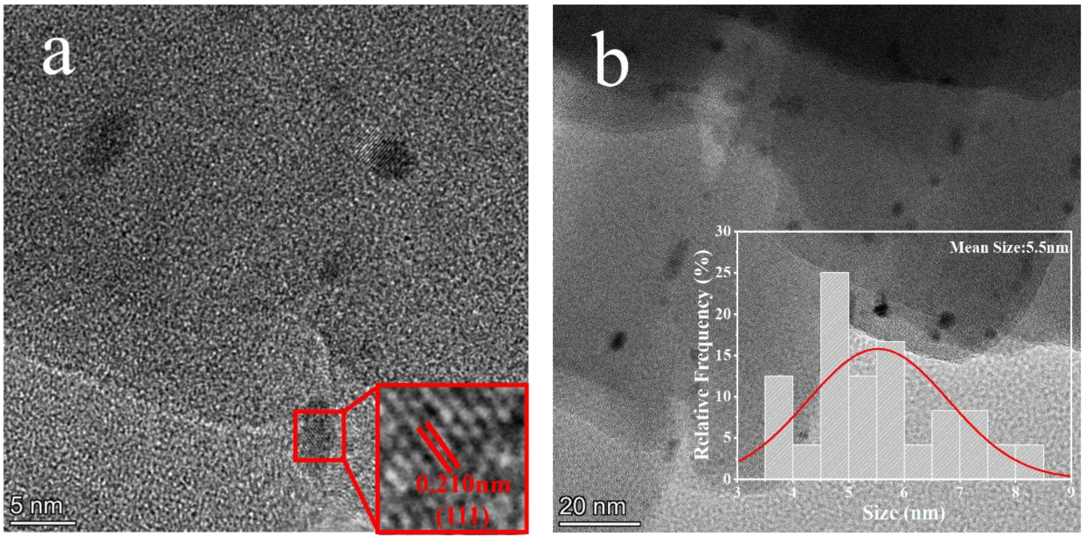

Figure S2. (a) HRTEM image of Ni nanoparticles in Ni/Nano-HL, and (b) STEM image of Ni/Nano-HL with particle size distribution.
